# Pharmacokinetic Properties and Systemic Safety of Vancomycin-Impregnated Cancellous Bone Grafts in the Treatment of Spondylodiscitis

**DOI:** 10.1155/2013/358217

**Published:** 2013-07-17

**Authors:** Konstantinos Anagnostakos, Katrin Koch

**Affiliations:** Klinik für Orthopädie und Orthopädische Chirurgie, Universitätsklinikum des Saarlandes, Kirrbergerstr. 1, 66421 Homburg, Germany

## Abstract

The aim of the present study was to investigate the local pharmacokinetic properties and the systemic safety of vancomycin-impregnated cancellous bone grafts in the treatment of spondylodiscitis. Between 2010 and 2012, 8 patients (5 females, 3 males, mean age 68.75 y.) were treated with this method. Local vancomycin concentrations reached median values of 179 *µ*g/mL (maximum 365 *µ*g/mL) on day 1, decreasing to 98 *µ*g/mL on day 3. The urine vancomycin concentrations showed similar pharmacokinetic properties as those locally determined. On day 1, median values were at 28.05 *µ*g/mL (maximum 287 *µ*g/mL). All serum vancomycin concentrations were in all cases and on every day below <2 *µ*g/mL. The median serum creatinine values were preoperatively 0.87 mg/dL, followed by 0.625 mg/dL, 0.705 mg/dL, and 0.835 mg/dL on day 7, 14, and 28, respectively. No cases of ototoxicity could be observed. At a mean follow-up of 16.5 [4–36] months no cases of reinfections or persistent infections could be seen. In conclusion, the implantation of vancomycin-loaded cancellous bone grafts is an effective option in the treatment of spondylodiscitis with a high infection eradication rate and no risk of any systemic toxicity. The pharmacokinetic properties can be easily monitored locally, in the urine and the serum.

## 1. Introduction

In the past years, an increasing number of studies about antibiotic-impregnated bone grafts (AIBGs) have been published with some very promising results [1]. AIBGs have been used in the treatment of infected total hip arthroplasties [2, 3] or infected tibia defects [4, 5] with infection control rates exceeding 90% [1]. 

With regard to spine surgery, there exists a single study reporting on the prophylactic use of AIBGs [6]. Borkhuu et al. reported a significant decrease in the infection rate after spinal surgery in children with scoliosis because of cerebral palsy after local use of AIBGs compared with a group where plain bone grafts were inserted (from 15% to 4%) [6].

Although the local use of AIBGs would make sense in the treatment of spine infections and especially of spondylodiscitis, there exist no studies up to date, to the best of our knowledge. In our department, we have started treating spondylodiscitis cases with AIBGs in 2010. Since this is a new indication field, we represent the opinion that the monitoring of the local and systemic pharmacokinetic properties of AIBGs should routinely occur until this treatment option is well established. Knowledge about the local and systemic release characteristics is an essential premise for assessing the length of sufficient antibiotic elution in vivo, efficiency of local infection eradication, but also for prevention of possible side effects in case of a systemic toxicity. Experiences with other antibiotic-loaded drug systems (acrylic bone cement) have shown that systemic side effects (e.g., acute renal or hepatic failure) might occur [7, 8] although these carriers are regarded to be safe with no risk of systemic adverse reactions [9, 10]. 

In the present retrospective study, we would like to report on the local and systemic pharmacokinetic properties of vancomycin-impregnated cancellous bone grafts in the treatment of the first eight spondylodiscitis cases we have treated.

## 2. Materials and Methods

Between 2010 and 2012 eight patients with a destructive spondylodiscitis have been treated with vancomycin-loaded cancellous bone grafts. There were five female and three male patients at a mean age of 68.75 [43–83] years. Three cases were localized in the thoracic, one thoracolumbar, and five in the lumbar spine. Five patients were treated with a one-stage and three with a two-stage protocol. Demographic data, microbiological findings, and treatment data are summarized in Table 1.

All patients were treated according to the same surgical protocol. Depending on the patient's comorbidities either a one- or a two-stage anterior-posterior stabilization was performed. Following the dorsal stabilization by internal fixation and drainage of an epidural abscess, if necessary, an anterior approach was utilized. The preferred approach was from the right side for the thoracic and from the left side for the lumbar cases. Intraoperative fluoroscopy was used to confirm the proper level of spondylodiscitis by placing a needle into the target disc space. Samples from the affected disc were sent for further microbiological and histopathological examination. After debridement of all infected, necrotic, and ischemic tissue, nucleotomy and (partial) vertebrectomy, the decision regarding the implant choice was made intraoperatively. Patients with large defects were treated with a vertebral body replacement system (VLIFT, Fa. Stryker, Germany) and AIBGs within. For those having too small defects for implantation of a vertebral body replacement system, an antibiotic-loaded femoral head was inserted in a press-fit technique instead. 

Commercially available, cancellous bone allografts (half femoral heads, Fa. Tutogen, Germany) were used in all cases. All patients were treated with vancomycin-impregnated bone grafts. Vancomycin was chosen due to its efficiency against gram-positive cocci which are regarded to be the most common causative organisms in cases of spondylodiscitis [11]. The antibiotic loading amount was determined according to the recommendations made by Witsø et al. [12]. The bone grafts were impregnated in vancomycin-containing solutions (concentration: 100 mg/mL, volume: 40 mL). The fluid volume was so defined that the grafts were completely covered by. The impregnation time was 20 min. In the cases treated with a vertebral body replacement system, the grafts were grinded to smaller bone chips and then inserted into the implant.

For assessment of the local pharmacokinetics, the vancomycin concentrations were determined postoperatively in the drain fluid on a daily basis. The drains were left in situ until less than 50 mL/days were produced. In the patients having a thoracic drainage the local concentrations could not be measured because this is a closed system and there was no possibility of taking samples without damaging the system. 

For assessment of the systemic pharmacokinetics and possible systemic side effects, vancomycin concentrations were determined in the urine. The urine concentration could be determined as long as a bladder catheter was placed in each patient. Serum vancomycin concentrations as well as creatinine values were measured on days 1, 3, 5, 7, 14, and 28 after surgery.

The vancomycin concentrations were measured by means of a fluorescence polarization immunoassay (FPIA) (AxSym-System, Fa. Abbot, Germany). The lowest detection limit of the method is at <2 *μ*g/mL. 

Postoperatively, all patients received a 6-week antibiotic therapy consisting of 4 weeks intravenous and 2-week oral administration. For avoidance of any interactions regarding the determination of the serum concentrations, vancomycin was not given intravenously in any patient.

## 3. Results

**Figure 1:**
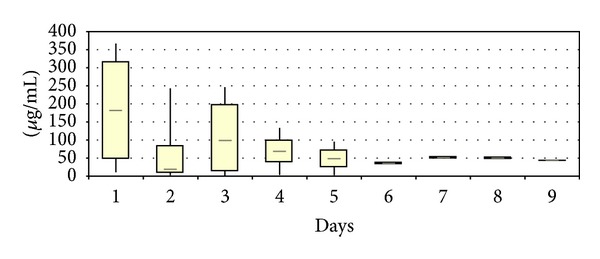
Local vancomycin concentrations after implantation of vancomycin-loaded bone grafts in the treatment of spondylodiscitis.

**Figure 2:**
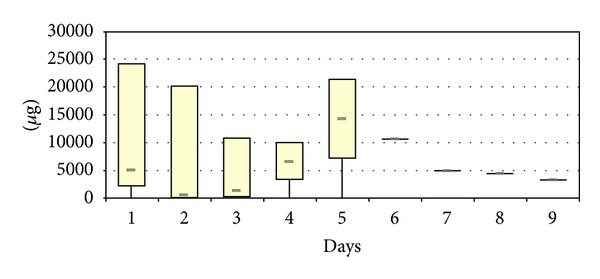
Total amount of locally released vancomycin after implantation of vancomycin-loaded bone grafts in the treatment of spondylodiscitis.

The local vancomycin elution could be determined in four patients with infections in the lumbar spine. Maximal concentrations were determined on day 1 with values reaching 365 *μ*g/mL (median 179 *μ*g/mL). In the following days, the eluted concentrations decreased rapidly, reaching median values of 98 *μ*g/mL on day 3. In one case (No. 1), the local concentrations could be measured over the first nine postoperative days with the concentrations being 41.9 *μ*g/mL on the last day (Figure 1). The total amount of locally eluted vancomycin was 75,000 *μ*g (median 5,100 *μ*g) on day 1, reaching median values of 1380 *μ*g on day 3. The maximum amount was measured in case No. 1 with 253,945 *μ*g vancomycin after 9 days (Figure 2).

**Figure 3:**
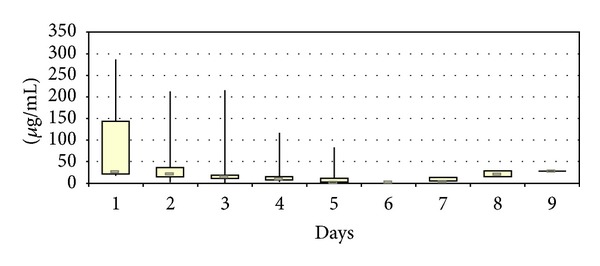
Urine vancomycin concentrations during the first 9 postoperative days concentrations after implantation of vancomycin-loaded bone grafts in the treatment of spondylodiscitis.

The urine vancomycin concentrations showed similar pharmacokinetic properties as those locally determined. Maximal concentrations could be seen on day 1 with values reaching 287 *μ*g/mL (median 28.05 *μ*g/mL). In the following days, the measured concentrations decreased rapidly, reaching median values of 16 *μ*g/mL on day 3, and 5.55 *μ*g/mL after one week. All determined concentrations were below those locally measured in every patient on every day (Figure 3).

All serum vancomycin concentrations were in all cases and on every day below the detection limit of the FPIA (<2 *μ*g/mL).

**Figure 4:**
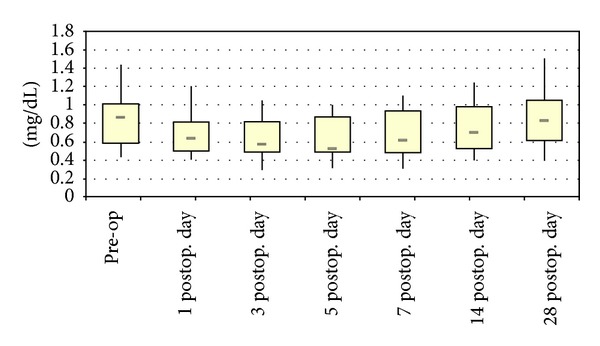
Serum creatinine values during the first 4 postoperative weeks after implantation of vancomycin-loaded bone grafts in the treatment of spondylodiscitis.

The median serum creatinine values were preoperatively 0.87 [0.43–1.42] mg/dL, followed by 0.64 [0.41–1.2] mg/dL on day 1, 0.575 [0.29–1.05] mg/dL on day 3, and 0.625 [0.32–1.1] mg/dL on day 7. After 2 and 4 weeks, the median values were 0.705 [0.4–1.24] mg/dL and 0.835 [0.4–1.5] mg/dL, respectively (Figure 4). One patient (No. 6) showed elevated serum creatinine values (1.42 mg/dL) preoperatively which remained stable over the four postoperative weeks (last determined values 1.5 mg/dL).

**Figure 5:**
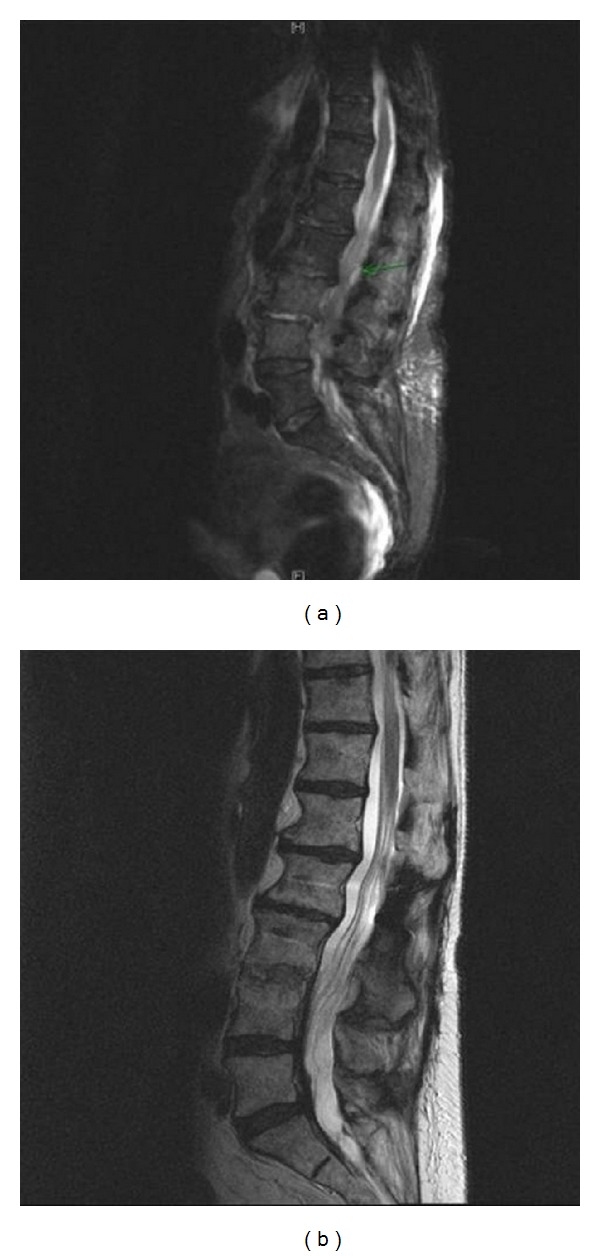
(a) Preoperative MR images of a 75-year-old female patient with a L3/4 MRSA-spondylodiscitis and intraspinal abscess (“green arrow”); (b) 18 months after surgery, a bony fusion with no signs of any infection is evident.

Following surgery, no cases of ototoxicity could be observed. At a mean follow-up of 16.5 [4–36] months no cases of reinfections or persistent infections could be seen (Figure 5).

## 4. Discussion

The aim of the present study was to investigate the local pharmacokinetic properties and the systemic safety of vancomycin-impregnated cancellous bone grafts in the treatment of spondylodiscitis. To the best of our knowledge, this is the first study which reports on this treatment option and tried to evaluate these parameters. Our results indicate that vancomycin can be locally released in high concentrations without any risk of a systemic toxicity. The infection eradication rate was 100% at a mean follow-up of 16.5 months.

Spondylodiscitis is the main manifestation of hematogenous osteomyelitis in patients aged over 50 years and represents 3–5% of all cases of osteomyelitis [11]. Predisposing factors include diabetes mellitus, advanced age, injecting drug use, immunosuppression, malignancy, renal failure, rheumatological disease, liver cirrhosis, previous spinal surgery, or distant focus of a present infection [11]. Although a wide range of causative pathogen organisms has been associated with spondylodiscitis, *S. aureus* still remains the predominant organism, accounting for half of nontuberculous cases [11].

Treatment may vary depending on the infection status, presence of spinal instability, and comorbidities of the patient [11]. Conservative treatment consists of antimicrobial therapy, physiotherapy, and immobilization. In case of compression of neural elements, spinal instability due to extensive bony destruction, severe kyphosis, or failure of conservative treatment, an operative management is indicated [11]. Some authors also consider the presence of an epidural abscess as an indication for surgery, even in the absence of neurological deficits [11]. A variety of surgical approaches exist including an anterior approach with decompression and insertion of an autologous bone graft or titanium cage or posterior decompression by laminectomy, for example, in case of an epidural abscess [11].

For local antibiotic treatment of the infection, several materials have been used including acrylic bone cement, collagen sponges as well as the combination of hydroxyl apatite and calcium sulphate [13]. The major advantage of these carriers is the local release of high antibiotic concentrations which vastly exceed those after systemic administration [13]. However, various advantages and disadvantages might be associated with these systems. Acrylic bone cement in form of beads can be optionally loaded with various antibiotics depending on the causative organism but has to be removed in a further surgery [13]. Commercially available collagen sponges elute >90% of the impregnated antibiotic within the first 48 hours and an organism-depending antibiotic impregnation is not possible [13]. The combination of hydroxyl apatite and calcium sulphate (PerOssal) offers the advantage of impregnating with several antibiotics without having a negative effect on the bone healing. Von Stechow and Rauschmann reported on the use of either gentamicin- or vancomycin-loaded PerOssal in the treatment of spondylodiscitis in 12 patients [14]. A bony fusion was achieved after 3–6 months. At a minimum follow-up of 1 year no reinfections could be observed.

In the present study the local antibiotic therapy consisted of vancomycin-impregnated cancellous bone grafts. The choice for this carrier as well as for this specific antibiotic for bone impregnation was made on the basis of several criteria. A bony fusion is always a major aim in the treatment of a spondylodiscitis and cancellous bone is an excellent tool for achieving this goal. Allografts were used instead of autologous cancellous bone (e.g., harvested from the iliac crest) because these grafts have a standardized size and, therefore the impregnation with antibiotics would allow for a standardized prediction of the antibiotic elution. Differences in the cancellous structure or the bone density of the harvested bone might have an influence on the antibiotic release and subsequently on the effectiveness of the local antibiotic therapy. The choice for vancomycin was made due to its efficiency against gram-positive cocci which are regarded to be the most common causative organisms in cases of spondylodiscitis [11]. Moreover, vancomycin possesses better elution characteristics from cancellous bone in comparison with other antibiotics [15, 16]. Furthermore, we decided for an impregnation with a single antibiotic and not with a combination of agents because it has been demonstrated that the amount of vancomycin eluted from vancomycin-netilmicin-loaded grafts was significantly reduced compared with those loaded only with vancomycin [17].

Knowledge about the elution kinetics of AIBG's is an indispensable premise for the successful planning and treatment of bone and joint infections. Several studies have investigated the pharmacokinetic properties of AIBGs in vitro and in vivo. Kanellakopoulou et al. determined the highest moxifloxacin concentrations exceeding 4,500 *μ*g/mL on the first day under experimental conditions [18]. Winkler et al. compared in vitro the elution kinetics of cortical and cancellous bone impregnated with vancomycin or tobramycin [15]. Highest initial vancomycin concentrations were meanly 20,900 *μ*g/mL and 5,700 *μ*g/mL, respectively (the difference was significant). The release of tobramycin was significantly lower than that of vancomycin. Buttaro et al. reported highest vancomycin concentrations of 1,400 *μ*g/mL from bone grafts used in the treatment of infected hip arthroplasties [2]. For the same surgical indication, Winkler et al. could measure mean vancomycin levels in the drainage fluid of 535 *μ*g/mL on the first postoperative day, declining to 400 *μ*g/mL on the third day [3].

Our results demonstrated highest concentrations of 365 *μ*g/mL with median values of 179 *μ*g/mL. The discrepancy to the data of the aforementioned studies might be explained by differences in the anatomic localization, impregnation method and time, antibiotic, used, or amount of the drainage fluid over the early postoperative period. However, the concentrations measured vastly exceed the minimal inhibitory concentration of the great majority of bacteria isolated in these infections.

A drawback of the determination of locally eluted antibiotic amounts is the fact that measurements can be only carried out as long as the drainage is left in situ. Any statements about the further local pharmacokinetics cannot be made. An indirect assessment of the local elution can be made by determining the antibiotic concentrations in the urine. A premise for the adequate interpretation of these data is an intact renal function with normal serum creatinine values. Otherwise, an accumulation of these concentrations with a prolonged renal elimination might occur. In the present study, all concentrations were below those locally measured in every patient on every day. All patients except for one had a normal renal function which remained normal through the entire treatment course over the first 4 postoperative weeks. These findings are extremely important. Locally released vancomycin amounts can be completely eliminated with no risk of systemic toxicity. As long as the urine concentrations do not exceed those locally determined there is no risk of a systemic accumulation and hence of a renal insufficiency or acute renal failure. These findings are confirmed by the serum vancomycin concentrations measured in the present study which were <2 *μ*g/mL in all cases on every day. Monitoring of these concentrations in the urine is easy and not expensive and should be recommended in our opinion until this rather new treatment option is widely established in clinical practice.

Literature data about the systemic safety of AIBGs are scarce. Witsø et al. studied the systemic concentrations after local insertion of netilmicin- and vancomycin-loaded bone grafts in a rabbit model [17]. In animals operated on with implantation of netilmicin-loaded bone, mean peak concentrations were 4.2 [3.7–4.7] *μ*g/mL 2-3 h postoperatively, whereas vancomycin could not be detected at all in serum. Winkler et al. could measure a mean postoperative serum level of vancomycin of 0.2 [0.0–1.8] *μ*g/mL on the first postoperative day [3]. In 37 cases the renal function did not show any remarkable changes postoperatively. Seber et al. determined the gentamicin urine levels after insertion of gentamicin-loaded xenografts in the treatment of osteomyelitis [19]. Mean levels at 24 h were 4 *μ*g/mL, falling below the effective level of 0.5 *μ*g/mL after 8 days. No cases of nephrotoxicity or ototoxicity could be observed.

In conclusion, the implantation of vancomycin-loaded cancellous bone grafts is an effective option in the treatment of spondylodiscitis with a high infection eradication rate and no risk of any systemic toxicity. The pharmacokinetic properties can be easily monitored locally, in the urine and the serum. 

## Figures and Tables

**Table 1:** Demographic data, microbiological findings, and treatment information of 8 patients suffering from a spondylodiscitis and being treated with vancomycin-loaded, cancellous bone grafts.

Patient	1	2	3	4	5	6	7	8
Age/gender	73/F	65/M	61/M	73/F	75/F	83/F	77/F	43/M
Spondylodiscitis localization	L3/4	L4/5	Th 6/7	Th 8/9	L3/4	L2/3	Th12/L1	Th10-11
Pathogen organism	n.o.i.	*S. hominis*	*S. epidermidis*	*E. cloacae*	MRSA	*E. coli*	n.o.i.	*S. aureus*
Surgical therapy	One-stage anterior-posterior stabilization	One-stage anterior-posterior stabilization	Two-stage anterior-posterior stabilization	One-stage anterior-posterior stabilization	Two-stage anterior-posterior stabilization	One-stage anterior-posterior stabilization	Two-stage anterior-posterior stabilization	One-stage anterior-posterior stabilization
Implant used	VLIFT + AIBGs	AIBGs	VLIFT + AIBGs	VLIFT + AIBGs	AIBGs	AIBGs	AIBGs	VLIFT + AIBGs
Intravenous antibiotic therapy	Meronem + daptomycin	Levofloxacin	Cefuroxime + rifampicin	Ceftazidime + refobacin	Rifampicin + daptomycin	Cefotaxime	Clindamycin	Cefuroxime
Oral antibiotic therapy	Levofloxacin	Levofloxacin	Linezolid	Levofloxacin	Linezolid	Ciprofloxacine	Ciprofloxacine	Cefuroxime
Follow-up [months]	13	27	16	12	36	12	12	4

M: male; F: female; n.o.i.: no organism isolated; AIBGs: antibiotic-impregnated bone graft; MRSA: methicillin-resistant *Staphylococcus* aureus.
